# Inferring Loss-of-Heterozygosity from Unpaired Tumors Using High-Density Oligonucleotide SNP Arrays

**DOI:** 10.1371/journal.pcbi.0020041

**Published:** 2006-05-12

**Authors:** Rameen Beroukhim, Ming Lin, Yuhyun Park, Ke Hao, Xiaojun Zhao, Levi A Garraway, Edward A Fox, Ephraim P Hochberg, Ingo K Mellinghoff, Matthias D Hofer, Aurelien Descazeaud, Mark A Rubin, Matthew Meyerson, Wing Hung Wong, William R Sellers, Cheng Li

**Affiliations:** 1 Departments of Biostatistics and Computational Biology and Medical Oncology, Dana-Farber Cancer Institute, Boston, Massachusetts, United States of America; 2 Departments of Medicine, Pathology, and Radiation Oncology, Brigham and Women's Hospital, Boston, Massachusetts, United States of America; 3 Departments of Medicine and Pathology, Harvard Medical School, Boston, Massachusetts, United States of America; 4 Department of Biostatistics, Harvard School of Public Health, Boston, Massachusetts, United States of America; 5 Department of Medicine, Massachusetts General Hospital, Boston, Massachusetts, United States of America; 6 Departments of Medicine and Medical Pharmacology, David Geffen School of Medicine, University of California Los Angeles, Los Angeles, California, United States of America; 7 Broad Institute of Harvard and MIT, Cambridge, Massachusetts, United States of America; 8 Department of Statistics, Stanford University, Stanford, California, United States of America; University of California San Diego, United States of America

## Abstract

Loss of heterozygosity (LOH) of chromosomal regions bearing tumor suppressors is a key event in the evolution of epithelial and mesenchymal tumors. Identification of these regions usually relies on genotyping tumor and counterpart normal DNA and noting regions where heterozygous alleles in the normal DNA become homozygous in the tumor. However, paired normal samples for tumors and cell lines are often not available. With the advent of oligonucleotide arrays that simultaneously assay thousands of single-nucleotide polymorphism (SNP) markers, genotyping can now be done at high enough resolution to allow identification of LOH events by the absence of heterozygous loci, without comparison to normal controls. Here we describe a hidden Markov model-based method to identify LOH from unpaired tumor samples, taking into account SNP intermarker distances, SNP-specific heterozygosity rates, and the haplotype structure of the human genome. When we applied the method to data genotyped on 100 K arrays, we correctly identified 99% of SNP markers as either retention or loss. We also correctly identified 81% of the regions of LOH, including 98% of regions greater than 3 megabases. By integrating copy number analysis into the method, we were able to distinguish LOH from allelic imbalance. Application of this method to data from a set of prostate samples without paired normals identified known regions of prevalent LOH. We have developed a method for analyzing high-density oligonucleotide SNP array data to accurately identify of regions of LOH and retention in tumors without the need for paired normal samples.

## Synopsis

A key event in the generation of many cancers is loss of heterozygosity (LOH) of chromosomal regions containing tumor suppressor genes, whereby one parent's version of the tumor suppressor is lost. As we develop a better understanding of the molecular mechanisms that generate different cancers, a description of the LOH events underlying these cancers is forming an important part of their classification. Generally, detection of LOH relies on comparison of the tumor's genome to the normal genome of the individual. Unfortunately, for many tumors, including most experimental models of cancer, the normal genome is not available. Therefore, the authors have developed a hidden Markov model-based method that evaluates the probability of LOH at all sites throughout the genome, based on high-resolution genotyping of only the tumor. They were able to achieve high levels of accuracy, specifically by taking into account the haplotype block structure of the genome. Application of this method to a set of 34 prostate cancer samples allowed the authors to identify the locations of the known and suspected tumor suppressor genes that are targeted by LOH.

## Introduction

Loss of heterozygosity (LOH) refers to change from a state of heterozygosity in a normal genome to a homozygous state in a paired tumor genome. LOH is most often regarded as a mechanism for disabling tumor suppressor genes (TSGs) during the course of oncogenesis [1,2]. Although LOH is often thought to result from copy-loss events such as hemizygous deletions, a large proportion of LOH results from copy-neutral events such as chromosomal duplications [3,4]. Analyzing LOH data across multiple tumor samples can point to loci harboring TSGs or identify subtypes of tumors with different somatic genetic profiles [5,6].

Single nucleotide polymorphisms (SNPs) are the most common genetic variation in the human genome and can be used to search for germline genetic contributions to disease. To that end, oligonucleotide SNP arrays have been developed to simultaneously genotype thousands of SNP markers across the human genome [7–9]. The density, distribution, and allele specificity of SNPs makes them attractive for high-resolution analyses of LOH and copy number alterations in cancer genomes [3,6,10–15].

Traditionally, LOH analyses require the comparison of the genotypes of the tumor and its normal germline counterpart. However, for cell line, xenograft, leukemia, and archival samples, paired normal DNA is often unavailable. Current generations of SNP arrays provide high enough marker density to make it feasible to identify regions of LOH by the absence of heterozygous loci (which we call inferred LOH), rather than by comparison to the paired normal. For example, the homozygosity mapping of deletions method was developed to use highly polymorphic microsatellite markers to identify regions of hemizygous deletion in unpaired tumor cell lines [16], and a simple method of inferring LOH using the product of the probability of homozygosity in neighboring SNPs was able to identify 80% of LOH in 10 K SNP array data from one sample [3,17]. SNP markers are less polymorphic than microsatellite markers, however, and the haplotype structure may render closely located SNPs dependent in their genotype calls. We hypothesized that a method that infers LOH with high accuracy would have to account for not only the varied heterozygosity rates of SNP markers, but also their varied intermarker distances, as well as genotyping errors and the interdependence of SNP alleles based on the haplotype structure of the genome.

We approached this problem by developing a hidden Markov model (HMM) to infer LOH. HMMs are appropriate for inferring the unobserved underlying states that give rise to an observed chain of data, using multiple sources of information. They have been used to model biological data in diverse applications such as sequence analysis [18–20], linkage studies [21,22], and array-comparative genomic hybridization [15,23]. SNP genotypes along a chromosome are chain-like and thus suitable for HMM analysis. The model we developed incorporates SNP intermarker distances, SNP-specific heterozygosity rates, and genotyping error rate. We investigated its ability to accurately identify regions of LOH in unpaired tumors, both at low genotyping densities (10,000 markers across the genome), where neighboring SNPs could be considered independent, and at high densities (over 100,000 markers across the genome), where linkage disequilibrium leads to dependencies between neighboring SNPs. We further applied this method to data from prostate cell lines, xenografts, and metastases lacking paired normals, to test its ability to identify known regions of prevalent LOH containing known and putative TSGs.

## Results

### A Basic HMM for Inferring LOH from Unpaired Tumor Samples

**Figure 1:**
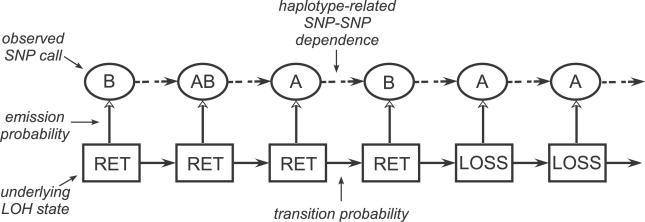
The Elements Included in the HMM for LOH Inference Unobserved LOH states (LOSS or RET) of SNP markers generate observed genotype calls via emission probabilities. The solid arrows indicate the transition probabilities between LOH states, and the dashed arrows indicate LD-induced dependencies between consecutive SNP genotypes.

The components of a HMM are the unobserved states, the observed measurements, the emission probabilities connecting these two, the transition probabilities between the unobserved states, and the initial probabilities of the states at the beginning of the chain (Figure 1). To infer LOH in unpaired tumor samples, we implemented a HMM with two unobserved states: loss (LOSS) and retention (RET) and the observed genotypes, reduced to homozygous (Hom; AA or BB), heterozygous (Het; AB), and “No Call.” We conceptualized that the observed genotypes are generated by the unobserved LOH states according to the emission probabilities of the HMM.

#### Emission probabilities.

For a SNP under the RET state, we observed Het calls with a probability equal to the heterozygosity rate of each SNP, which we estimated from normal samples (see Materials and Methods). For a SNP under the LOSS state, we always observed a Hom call unless a genotyping or SNP mapping error has occurred. Since genotyping errors occur at a rate < 0.01 [7], we set the emission probability of a Het call under the LOSS state to 0.01. The emission probability of the Hom call at a SNP is one minus the emission probability of the Het call at the SNP. A SNP with “No Call” could have had either an unobserved Hom or Het call, and is therefore emitted with a probability of 1 regardless of its underlying LOH state. As a result, a “No Call” does not bias the inference toward either LOSS or RET.

#### Initial probabilities.

These probabilities, denoted by *P*_0_(RET) and *P*_0_(LOSS) = 1 −*P*_0_(LOSS), specify the probabilities of RET and LOSS for the p-terminal marker on a chromosome. They also specify the probabilities of the RET and LOSS states for any marker, if no other information exists for that marker. Assuming Het markers are observed in regions of LOSS only as a result of genotyping or mapping errors, the observed proportion of Het markers in a tumor sample is *P*_0_(RET) × average heterozygosity rate + *P*_0_(LOSS) × SNP error rate. As the SNP error rate is small the second term can be omitted. Therefore we estimated *P*_0_(RET) by dividing the proportion of Het markers by the average heterozygosity rate of SNPs in the population.

#### Transition probabilities.

These probabilities describe the dependence between the LOH states of adjacent markers. For any two adjacent SNP markers, we first defined *θ* as the probability that the state of the first marker does not inform the state of the second (i.e., that the LOH state of the second marker is distributed according to the initial LOH state probabilities). Empirically, nearby markers tend to have the same LOSS or RET state, while distant markers do not. To capture this observation we calculate *θ* using an increasing function *θ* = (1−*e* ^−2*d*^), where *d* is the physical distance (in the unit of 100 megabases [Mb] ≈ 1 morgan) between the two adjacent SNP markers. With probability 1 − *θ,* the two markers have the same LOH state. Therefore, the marker-specific transition probabilities of the second marker being LOSS given the LOH state of the first marker are:


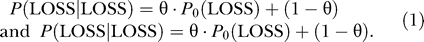


**Figure 2:**
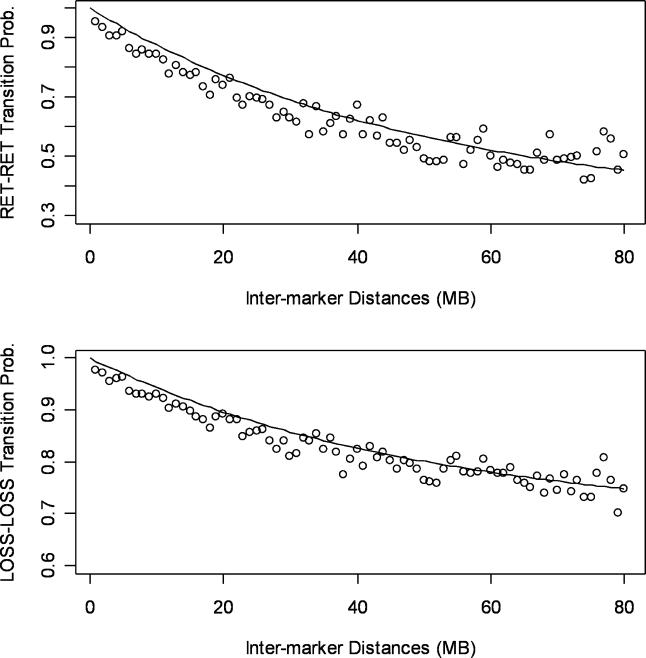
Comparison of Predicted to Empirically Determined LOH Transition Probabilities Empirically determined transition probabilities (circles) between RET loci (top graph) and LOSS loci (bottom graph) are compared to those predicted by Equation 1 (black lines).

The probability of RET at the second marker is one minus these two probabilities. This transition probability model is the same as those used in the “instability-selection” model for LOH analysis [24,25], and is reminiscent of Haldane's map function in linkage analysis [22]. We used a fixed scaling of *d,* instead of estimating it as in the instability-selection model, but this does not affect the method performance (see below). In addition, the empirical transition frequencies estimated from observed LOH calls in paired normal and tumor samples agreed well with the transition probabilities estimated by this model (Figure 2).

#### Inferring LOH states.

The HMM and these emission, initial, and transition probabilities specify the joint probability of the observed SNP genotypes and the unobserved LOH states in one chromosome of a sample. We applied the forward-backward algorithm [20] separately to each chromosome of each sample to obtain the LOSS probability for each SNP given all the genotype data on the chromosome. Alternatively, the Viterbi algorithm can be used; we found this gave similar LOH calls in 98.8% of SNPs (unpublished data). LOSS and RET calls were made using the least stringent threshold: LOSS if the SNP has a probability of LOSS greater than 0.5 and RET otherwise.

An alternative inference method for HMM is the Baum-Welch algorithm [20], which estimates the model parameters together with unobserved LOH states by an iterative procedure. We chose not to use this algorithm, as there are many parameters in the model (e.g., the transition probabilities depend both on the LOH states and on the distance between adjacent markers), but relatively few data points at each SNP position to estimate these parameters. This could lead the Baum-Welch algorithm to converge to local maxima when estimating optimal model parameters. Instead, we set biologically reasonable model parameters as above, with smooth transition probabilities that agreed with the observed data (Figure 2). In addition, we showed that the model inference accuracy is robust to the specified parameters in the initial, emission, and transition probabilities (see below).

#### The performance of the basic HMM.

**Figure 3:**
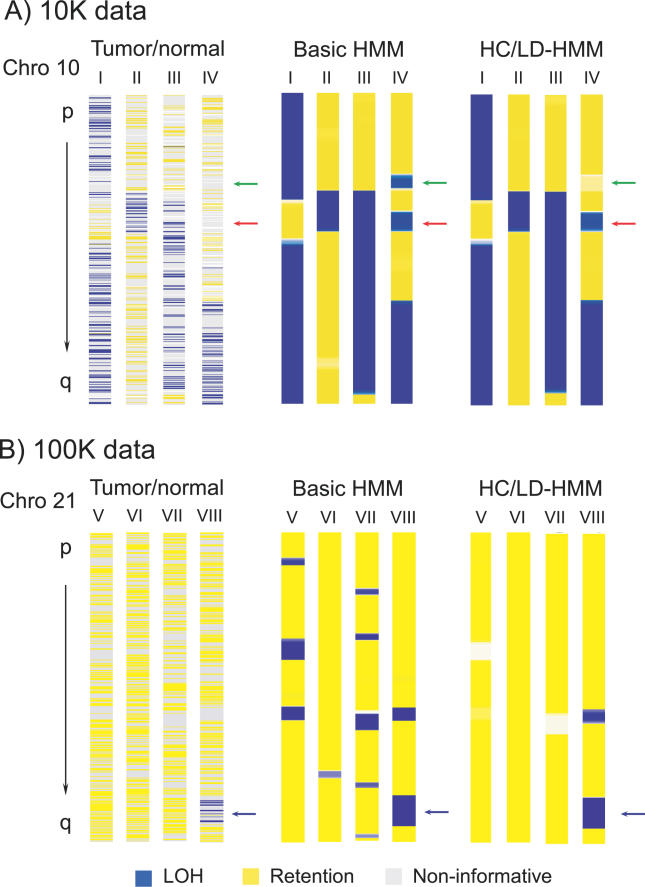
Comparison of LOH Inferred from Unpaired Tumors to LOH Observed in Tumor/Normal Pairs (A) Results from 10 K SNP array data. Each column represents a sample, with SNP markers from Chromosome 10 displayed from the p terminus (top) to the q terminus (bottom) (not all markers are displayed at this resolution). Tumor/normal observations (left) represent direct comparisons of tumor to normal genotypes. Here, SNP markers observed as having undergone LOH are indicated in blue, retention is shown in yellow, and noninformative SNPs are indicated in grey. Inferences from unpaired tumor data represent the probability of each SNP having undergone LOH, as made by the basic HMM (center) and HC/LD-HMM (right). Here, a high probability of LOH (LOSS) is also indicated in blue, a high probability of retention (RET) is indicated in yellow, and indeterminate SNPs with an almost equal probability of either state are indicated in white. Occasionally, regions that are noninformative in the tumor/normal comparison are falsely inferred as LOH by the basic HMM in the unpaired data (red arrows); some of these false regions are corrected by the HC/LD-HMM (green arrows). (B) Results from 100 K SNP array data, shown as in (A). Data from Chromosome 21 are shown to highlight the detection of false LOH in the analysis of unpaired tumor data, and are not representative of the frequency of true LOH events in this sample set. Almost all regions falsely inferred as LOH by the basic HMM are correctly inferred by the HC/LD-HMM. The blue arrows indicate a region of true LOH, which is correctly identified by both the basic and HC/LD-HMM.

We compared tumor-only inferred LOH to the observed LOH calls determined by paired analysis of tumor and normal genotypes, using 10 K SNP array data from autosomes of 14 lung and breast cancers and EBV-transformed normal cell line pairs (Figure 3A) [15]. Here, 17,511 of 17,922 markers observed as LOSS in tumor/normal pairs were called LOSS in unpaired tumors by the HMM (for a sensitivity of 97.7%), and 15,962 of 16,364 markers that were observed as RET in tumor/normal pairs were called RET in unpaired tumors (for a specificity of 97.5%) (Table S1A).

This initial analysis does not, however, account for the SNPs that are homozygous in both tumor and the paired normal, and thus are noninformative. A string of such homozygous SNPs may be falsely called LOSS in the HMM analyses of unpaired tumors, but not accounted for in the above comparison of observed and inferred LOH states (the red arrows in Figure 3A point to two examples). To estimate the extent of such potentially falsely inferred LOH, we assigned an LOH state (LOSS or RET) to those noninformative markers for which the first informative marker on either side had the same LOH state. For example, a noninformative marker would be assigned a LOSS state if the nearest flanking informative markers were both in the LOSS state. In this analysis, the noninformative makers assigned a RET state were falsely inferred as LOSS at a rate of 6.8% (10 K array) (Table S1A). Not surprisingly, false inferences of RET were rare, occurring at a rate of 0.3%. Taking into account the noninformative markers in this way, the overall sensitivity remained high at 99.1%, but the specificity dropped to 94.3%. As an alternative approach to the use of flanking markers, we also inferred the LOH states of uninformative markers through the application of an HMM to the paired tumor/normal data, with nearly identical results (Protocol S1 and Table S2).

### Linkage Disequilibrium Attenuates the Performance of the Basic HMM at High SNP Density

**Figure 4:**
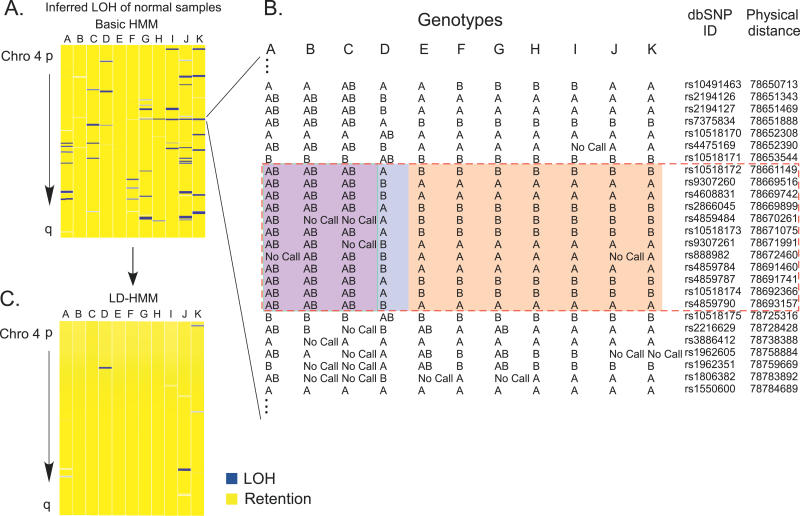
Accounting for LD by the LD-HMM Significantly Reduces False LOH Inferences from Data Obtained at High Marker Density (A) Inferences from the basic HMM applied to 100 K SNP array data are shown for Chromosome 4 in normal samples. Data are shown as in Figure 3. (B) The genotypes of one region of falsely inferred LOH reveal a region of linkage disequilibrium (dashed red box), also identified by the HapMap project. The sample in column “D” contains one haplotype, the samples in columns “E” through “K” contain another haplotype, and the samples in columns “A” through “C” are heterozygous. (C) Improved LOH inferences after application of the LD-HMM.

With these methods in place, we next applied the basic HMM to 100 K SNP array data from two prostate cancers and two lung cancer cell lines along with paired normal DNA, which were not included in the 10 K dataset. Here, the number of noninformative regions inferred as LOSS increased significantly (Figure 3B). When noninformative marker status was assigned as above, many of these regions were deemed false regions of LOSS, and the specificity of the HMM decreased to 92.2% (Table S1B). Furthermore, when 100 K SNP array data derived from normal samples alone were analyzed, the basic HMM identified multiple regions of LOH that by definition are false (Figure 4A). We found that this occurred because, at high SNP densities regions of linkage disequilibrium (LD) are probed multiple times, resulting in strings of homozygous SNPs. Specifically, if both parental chromosomes share the same haplotype, an extended stretch of homozygous genotypes will result. Therefore the assumption, inherent in the basic HMM, of independence between allele calls of adjacent or nearby SNPs becomes erroneous, leading to false inferences of LOH. An example is shown in Figure 4B, where the examination of an area of false LOH reveals the presence of a region of LD (dashed red box; also identified in the HapMap Project, available at: http://www.hapmap.org).

### HMM and Haplotype Correction that Incorporate LD Information

As indicated above, within a region of LD, the observed genotype of any marker depends not only on the underlying LOH state, but also on the genotypes of the adjacent markers (i.e., the two markers are dependent in genotype, indicated by the broken arrows in Figure 1). Here we account for many of these LD-induced SNP dependencies using an extension of the basic HMM (referred to herein as the linkage disequilibrium HMM or LD-HMM).

#### Expanded states and emission probabilities.

We use the same observed Het and Hom genotypes of the tumor sample as in the basic HMM, but expand the unobserved LOH states for a SNP marker from the previous two states (LOSS or RET) to four states: Hom LOSS, Het LOSS, Hom RET, and Het RET. Here Hom and Het represent the SNP marker's genotype in the unobserved normal sample. For example, “Hom LOSS” indicates that the SNP is homozygous in normal and LOH in tumor. The state “Hom LOSS”, “Het LOSS”, and “Hom RET” will result in homozygous genotype calls in the tumor unless genotyping or mapping error occurs, so the emission probability of the Hom genotype from these three states is set to (1 – SNP error rate). The state “Het RET” will result in a heterozygous SNP call in the tumor unless a genotyping or mapping error happens, so the emission probability of the Hom genotype from this state is set to the SNP error rate. The emission probability of the Het genotype is 1 minus that of the Hom genotype.

#### Transition probabilities.

The transition probabilities now reflect both the probability of a state change from RET to LOSS (LOH state), and a state change from Het to Hom (genotype state). We estimated genotype dependencies as the probability, for each SNP marker, of the next adjacent SNP marker toward q-arm being Hom (or Het), given the current SNP marker being Hom (or Het), in a reference set of normal samples (see Materials and Methods). We denoted these conditional probabilities for SNP *i* by P(*U_i_*_+1_ = Hom|*U*_i_ = Hom) and P(*U_i_*_+1_ = Het|*U*_i_ = Het). P(*U_i_*_+1_ = Het|*U*_i_ = Hom) and P(*U_i_*_+1_ = Hom|*U*_i_ = Het) are one minus the previous two probabilities, respectively. When there were not enough data to estimate these probabilities at a marker, the SNP-specific heterozygosity rate was estimated from the reference set and used as the unconditional probabilities—e.g., replacing P(*U_i_*_+1_ = Het|*U_i_* = Hom) by P(*U_i_*_+1_ = Het), the heterozygosity rate of marker *i* + *1*. Next, we built the transition probabilities by combining the above genotype dependence probabilities with the probability of an LOH state change. We denote the underlying LOH state of marker *i* by *U_i_V_i_,* where *U_i_* is either Hom or Het and *V_i_* is either LOSS or RET. Suppose the current SNP *i* is in the “Hom LOSS” state while the next SNP *i* + *1* is in the “Het RET” state. For this to happen, two independent events must occur: a homozygous genotype is followed by a heterozygous genotype in the normal with the probability P(*U_i_*_+1_ = Het|*U_i_* = Hom) estimated as above, and the LOH state changes from LOSS to RET in the tumor with the probability *P*(*V_i_*_+1_ = RET|*V_i_* = LOSS) as specified in the transition probability of the basic HMM. The transition probability from “Hom LOSS” to “Het RET” is then the product of these two probabilities. In general, the transition probability of going from LOH state *U_i_V_i_* to *U_i+1_V_i+1_* is *P*(*U_i_*_+1_ *V_i_*_+1_|*U_i_V_i_*) = *P*(*U_i_*_+1_|*U_i_*)*P*(*V_i_*_+1_|*V_i_*).

#### Inferring LOH states.

With the addition of the initial probabilities (which are the same as the basic HMM), the HMM parameters were fully specified and the forward-backward algorithm was used to obtain the probability of the LOH state being LOSS (either “Hom LOSS” or “Het LOSS”) for every SNP, given all the observed SNP calls along one chromosome of a tumor sample. Application of the LD-HMM to the 100 K dataset of normals, in place of the basic HMM, reduced the frequency of loss calls from 4.7% to 1.5% of markers (Figure 4C). Likewise, application of the LD-HMM to the 100 K training dataset improved the specificity of LOSS calls from 92.2% to 97.4%, while decreasing the sensitivity only from 99.8% to 99.6% (Table S1).

#### Empirical haplotype correction.

We posited that the remaining regions of falsely inferred LOH resulted from three specific deficiencies of the LD-HMM. First, regions of LD might be present in a relatively small subset of patients [26]. Across the population as a whole, the genotypes of the neighboring SNPs within these LD regions correlate only weakly, and thus are not taken into account by the LD-HMM. Second, LD may happen between markers that are not immediately adjacent. Finally, in the LD-HMM, the dependency information among SNPs are estimated for the reduced genotype calls (Hom/Het) rather than from real genotypes. To try to address these concerns we also developed an empirical haplotype correction method, in which we applied a computational correction to the inferred LOH regions from either the basic or LD-HMMs (herein referred to as HC-HMM and HC/LD-HMM, for the haplotype-corrected versions of the basic and LD-HMMs). For every putative LOH region called by HMM (LOH probability > 0.5 for all the SNP markers in the region but ≤ 0.5 for the SNPs at the boundaries of the region; containing mostly Hom SNP genotypes), we determine whether over 95% of the homozygous markers in this region in an unrelated normal reference sample are genotypically identical to the LOH region of the tumor sample. If this is the case, then the tumor sample is likely to share its haplotype structure with the reference sample in this region. Thus, homozygosity is likely due to LD rather than LOH, and the region is removed by setting the LOH probability of all the SNPs in the region to the LOH probability of the SNP marker just outside the bottom boundary of the region. This haplotype correction further improved specificity over the LD-HMM, in both the training 10 K and 100 K datasets, without significant loss of sensitivity (Figure 3 and Table S1A and S1B).

### The HC/LD-HMM Infers LOH with High Accuracy in a 100 K Validation Dataset

To validate these results, we extended the analysis to a set of 100 K data obtained from two lung cancer cell lines and six gliomas with paired normals, that had not been used in any of our prior analyses. Here, the sensitivity and specificity of the HC/LD-HMM were 98.7% and 99.3%, respectively (Tables 1 and S1C). Compared to the basic HMM, the HC/LD-HMM led to a greater than 8-fold reduction of potentially false LOH inferred at noninformative markers in the 100 K data, but remained highly sensitive for real LOH events. Interestingly, once the haplotype block structure of the human genome is taken into account, the performance of HMM-based inferred LOH is better for 100 K data than 10 K data, presumably due to the denser SNP coverage of the 100 K array.

**Table 1:**
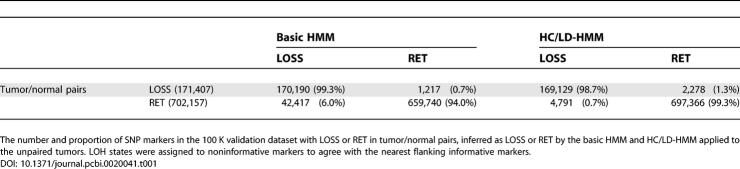
Sensitivity and Specificity of the Basic HMM and HC/LD-HMM

### Effect of Ethnicity

Given the importance of taking into account haplotype block structure, which is known to vary between ethnic groups [26], we sought to delineate the effect of mismatching the ethnicities of tumor and reference samples. To that end, we replaced the 60 Caucasian reference samples (the CEPH set) with, in turn, 89 East Asian samples (the JHC set), and 60 African samples (the YOR set) [27]. With each set, we then retested the basic HMM against our training set of tumor samples (Table S3). In each case, the sensitivity of the method remained almost unchanged, but the specificity dropped somewhat: from 97.4% in the case of the appropriately matched CEPH samples, to 97.0% and 93.8% in the cases of the JHC and YOR samples, respectively. The LD-HMM relies on estimates of genotyping dependencies between neighboring SNPs, which in turn are determined by the size of regions of LD in the reference datasets. Therefore, the similarity between results using the CEPH and JHC samples may reflect the similar size of regions of LD in the two groups, whereas the poorer specificities using the YOR samples as a reference may be due to the much smaller regions of LD in that group [27].

Conversely, the haplotype correction method relies on the ability to match specific haplotypes present in the tumors to those same haplotypes in the reference set. One might expect, therefore, that use of the JHC samples as the reference set for haplotype correction would result in poorer specificity than use of a Caucasian reference set. That is in fact the case, with the specificity of the HC/LD-HMM rising to only 98.3% when JHC samples were used for the haplotype correction, rather than the 99.0% when Caucasian samples were used (Table S3).

### LOH Inference Is Robust to Model Parameter Specifications

The methods described above rely on the empirical estimates of a number of the parameters used in the initial, emission, and transition probabilities of the HMM. To assess whether the tumor-only inference methods were unduly influenced by these estimates, we tested the performance of the basic and LD-HMMs as we varied these parameters. Specifically, the accuracy of the model results, as judged against observed LOH in the paired tumor/normal data, changed by less than 0.3% as the SNP error rate was varied from 0.1% to 1% (10 K array). Moreover, when the SNP-specific heterozygosity rates were replaced by an average heterozygosity rate that was varied from 0.1 to 0.5 (10 K array) or from 0.1 to 0.27 (100 K array), the accuracy of the model results changed by less than 5% and 0.5%, respectively. Likewise, use of 60 versus 89 reference samples (from the JHC reference set) affected model accuracy by less than 0.1%. We also found that varying the scaling factor *d* from 50 Mb to 200 Mb changed the LOH inferences of only 2% of SNP markers. These results suggest that the basic and LD-HMMs should be able to provide accurate LOH inferences in datasets that have different error rates, heterozygosity rates, or LOH-retention transition frequencies from the sample sets presented here, and that the set of reference samples used for determination of allele frequencies and dependencies need not be larger than 60 individuals.

### Why an HMM?

If the HMM is robust to parameter specifications, the question naturally arises, Why institute an HMM-based approach that requires these parameters, rather than a more simplistic approach? The most obvious simple approach is to calculate, for all *n* and in the reference set of normal samples, the probability with which a window of *n* homozygous SNPs occurs. Using this, one could determine a threshold number *t:* If a tumor contained a string of more than *t* homozygous SNPs, that region is likely to be suffering LOH.

We applied such an approach (called NumHom) to our training set of 100 K data, and found that it does not match the specificity of the HC/LD-HMM for thresholds at which reasonable sensitivities are reached (Table S4). Inspection of the data (Figure S1) reveals that, as with the basic HMM described above, much of this lack of specificity occurs due to unexpectedly long stretches of homozygous SNPs in particular regions, due to linkage disequilibrium. Therefore, we applied the haplotype correction method (described above) to the output of the NumHom method, to remove those regions of putative LOH that matched the genotypes of equivalent regions in our reference normal dataset. As expected, specificities increased markedly (Table S4), but at the cost of unacceptable decreases in sensitivity. Again, inspection of the data (Figure S1) revealed the reason: large regions of LOH were divided into subregions by the NumHom method, due to occasional intervening heterozygous SNPs. Those subregions often match genotypically their counterparts in the reference dataset, and are incorrectly removed. Conversely, the HC/LD-HMM allows for occasional heterozygous SNPs (representing genotyping errors, possibly exacerbated in some tumor samples by small amounts of contamination with normal cells) within large regions of LOH. As these large regions of LOH are not split into subregions by the HC/LD-HMM (Figure S1), the haplotype correction is applied to the overarching large region of LOH, which is retained.

The value of this HMM-based approach appears to be that it can straightforwardly integrate multiple sources of information, including SNP-specific heterozygosity rates, haplotype block structure, and genotyping error rates, to generate a local probability of LOH. It appears that each of these sources of information is necessary to obtain the highest sensitivity and specificity. Undoubtedly, other approaches may be devised to integrate these sources of information, but such approaches are likely to have a similar complexity to the HMM-based methods.

### Resolution of the HC/LD-HMM

The above analyses suggest that the HMM-based methods are robust for inferring LOH on a per marker basis. We next asked whether the HC/LD-HMM was equally effective in detecting regions of LOH and whether detection of such regions was influenced by their size. To this end, we compared the ability of the tumor-only LOH analysis to identify LOH regions observed from comparing paired normal and tumor samples (Table 2). Here, we define a LOH region in the paired analysis as containing at least three LOH markers with any number of intervening noninformative markers, and with boundaries defined in each direction by two consecutive retention markers. We considered such a region to have been “identified” by the tumor-only method, if that method inferred a probability of LOH higher than 0.5 for more than 90% of the SNP markers in the region. In the 100 K datasets (both training and validation), the majority of regions of LOH observed in paired tumor/normal analysis are over 3 Mb or are covered by at least 100 SNP markers, and more than 95% of these regions were identified using the unpaired analysis. Not surprisingly, smaller regions of LOH were detected less frequently. Overall, 80.8% of the regions of LOH identified in tumor/normal pairs were also identified in unmatched tumors in the 100 K SNP data (Table 2). A similar analysis of the 10 K data suggests higher sensitivity for smaller regions, apparently due to fewer such regions being identified by the tumor/normal paired analysis (Table S5).

**Table 2:**
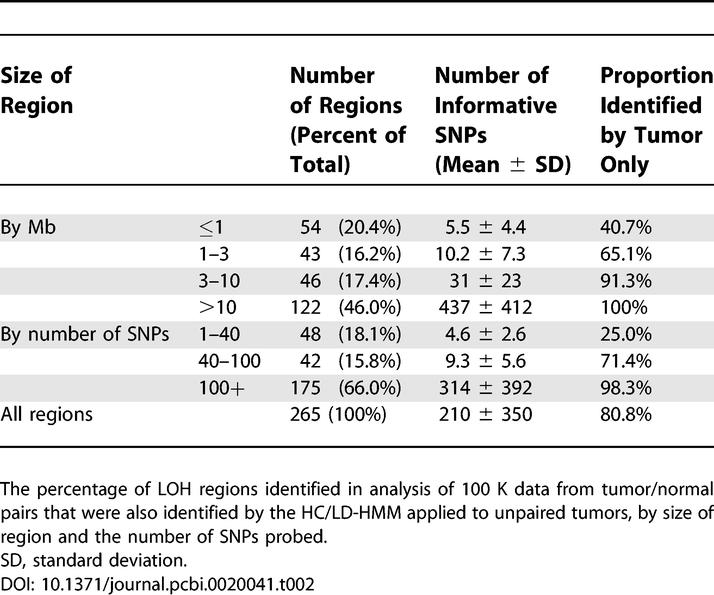
Sensitivity of the HC/LD-HMM by Size of LOH Region

### Integrating with Copy Number Analysis to Distinguish Allelic Imbalance

**Figure 5:**
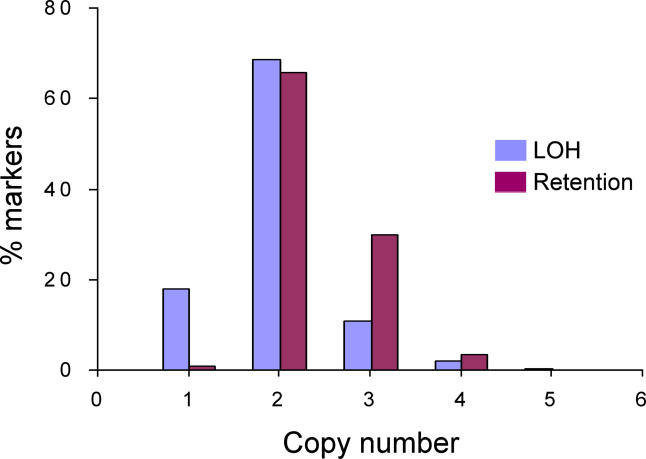
Correspondence between LOH and Copy Number For each inferred copy number (*x*-axis), the proportion of SNP markers (*y*-axis) observed in the 10 K dataset of tumor/normal pairs to have undergone LOH (blue) or retention (red) are shown.

As mentioned in the introduction, LOH arises due to complete loss of one allele through hemizygous deletion (copy loss) or through gene duplication (copy neutral). On the other hand, heterozygous loci can erroneously be assigned a homozygous genotype in settings of allele specific amplification (allelic imbalance). This will occur whether or not LOH is determined using paired normals, and may present paradoxical results, with recurrently amplified oncogenes seen as potential TSGs. To address this issue we determined the copy number at each SNP locus using the probe level signal intensity data [15] and correlated the results with the LOH analysis. We found that among the observed LOH from normal/tumor pairs or the inferred LOH from unpaired tumors (using the basic HMM), about 70% of SNPs have copy number 2 (copy neutral LOH), 20% have copy number 1 (copy loss LOH), and 10% have copy number 3 or above (amplification with possible allelic imbalance) (Figure 5). In contrast, among SNPs with observed retention from normal/tumor pairs or inferred retention from unpaired tumors, a lower percentage of markers have copy loss, and a higher percentage have amplifications (Figure 5). The combined LOH and copy number analysis can thus distinguish true LOH from those caused by amplification or allelic imbalance, which can be excluded from downstream LOH analysis. In addition, the copy number analysis can be used to distinguish LOH events caused by copy neutral gene conversion and copy number loss (Figure 5) [10,15]. In short, the vast majority of the regions of LOH detected using SNP arrays either by paired or unpaired analysis arises from copy neutral or copy loss events. Interestingly, the high frequency of copy-neutral LOH observed in these samples and others [3] suggests that LOH and copy number analyses provide independent sets of information pointing to TSGs.

### Common LOH Regions in a Set of Prostate Cancer Samples

**Figure 6:**
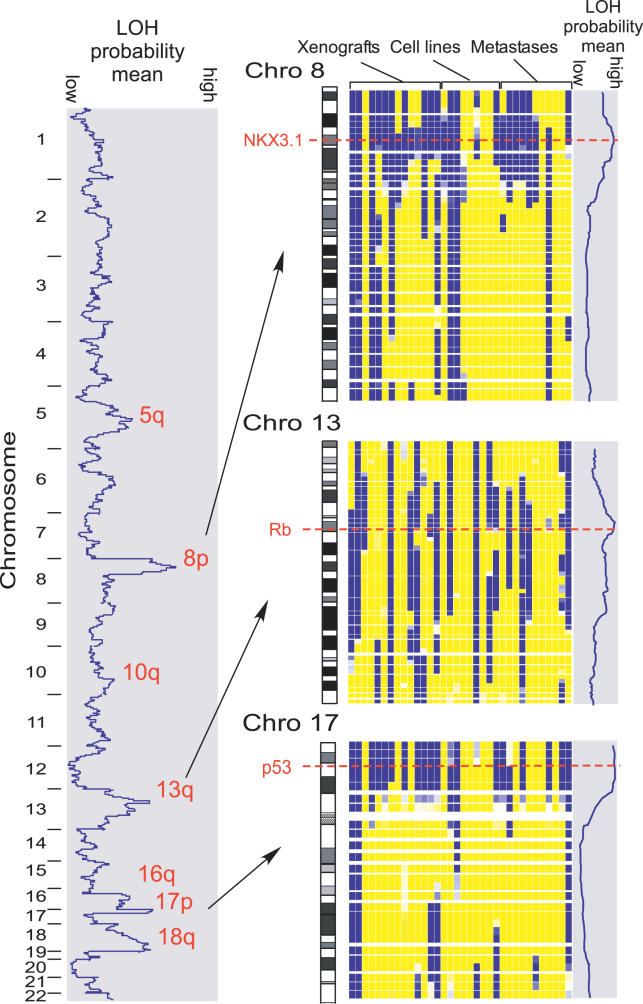
Inferred LOH in Prostate Cancer Samples Identifies Regions of LOH Known to Be Frequent in Prostate Cancer The mean LOH probability across 34 prostate cancer samples is plotted along the left for all chromosomes. Peak regions of LOH are noted, and data from Chromosomes 8, 13, and 17 are highlighted on the right. These data are displayed as in Figure 3. Note that in this view, SNPs are visualized proportionally to physical distance along the chromosome, and most SNPs are not projected due to proximity to their neighbors. The red dotted lines indicate the approximate chromosomal positions of putative TSGs.

Models of human cancer including xenografts and cell lines rarely are accompanied by paired normal samples. The utility of such models may be enhanced if we can ascertain the patterns of LOH in such models and relate them to those seen in actual human tumors. To this end, we next asked whether the HC/LD-HMM could detect regions of common LOH using 11 K SNP array data from 34 prostate cell lines, xenografts, and metastases where the corresponding normal DNA was unavailable (RB, unpublished data). We first scored each SNP by averaging the probability of LOH over all 34 samples (Figure 6, blue curves). The regions with the highest average probability of LOH correspond to known regions of frequent LOH, with several known and postulated TSGs lying in or near the regions with peak LOH scores (Figure 6 and Table S6). These data suggest that the tumor-only LOH and copy number inference can be used to detect regions of true LOH where paired samples are not available.

## Discussion

We have developed an HMM-based method to infer the probability of LOH events from tumor samples without matched normals. The method utilizes several sources of information, including intermarker distances, SNP genotyping and mapping error rates, and haplotype information. LOH inferences using only tumor samples agree well with LOH patterns determined by analysis of tumor/normal pairs in two different array types (10 K and 100 K), three different tissue types (lung, glioma, and prostate), and in both cell lines and tumors, in test and in validation datasets. The inferences are robust to model parameter specifications. LOH is resolved to about 3 Mb or 100 SNPs in 100 K array data. This method makes it feasible to use SNP array technology to map LOH in tumor samples for which normal DNA is unavailable. Given that genotyping paired normal samples constitutes up to half the cost of LOH mapping experiments, this method also makes it feasible to perform these experiments at a much lower cost per sample, at the expense of slightly reduced accuracy.

One advantage of a model-based approach over the existing tumor-only LOH inference methods [3,16] is its extensibility. The basic HMM was developed using average heterozygosity rates, but readily extended to incorporate the SNP-specific heterozygosity rates and haplotype information as they became available. In addition, rather than making definitive calls the algorithm infers the probability of LOH at each marker of a sample. This SNP-specific probability can then be used in further downstream analyses, such as identifying regions of shared LOH and sample clustering [5,24,28]. For example, a high probability of LOH across many samples can indicate potential TSGs (Figure 6). The HMM approach can also be used to infer LOH probabilities for paired normal and tumor samples (see Protocol S1), unifying the LOH analysis for paired tumor/normal and unpaired tumor samples.

At higher SNP densities, where the haplotype structure of the human genome becomes relevant, an approach that considers the dependence among multiple SNPs in a region of LD is necessary in addition to the LD-HMM. We used a haplotype correction that compared regions of inferred putative LOH to a set of reference normal samples to reduced false LOH inference. This method works best if the reference samples have similar haplotypes to the tumor sample. Use of reference samples from a different ethnic group tends not to decrease the sensitivity of the method, but can substantially decrease its specificity.

False designation of regions of LOH due to allelic imbalance may lead to paradoxical results, with recurrently amplified oncogenes seen as potential TSGs. SNP arrays, by providing signal intensity along with genotyping data, allow such regions to be identified. We can thus integrate these data to exclude regions of putative LOH with high copy numbers as likely due to allelic imbalance. At the interpretive level, our finding that LOH is often copy-neutral suggests that LOH and copy loss should be considered independently when predicting the presence of a TSG, and may best be used in conjoined analyses.

The ability to identify regions of LOH in tumors without paired normal DNA allows LOH mapping in the many model systems lacking paired normal DNA, including cell lines and xenografts. As such model systems are the platform for experiments aimed at understanding the biology of human tumors, it is critical that we understand their genetic relationship to real human tumors. As an example, among the prostate cancer samples, LOH at the *NKX3.1* locus is more prevalent among real tumors and xenografts than among cell lines, LOH at the *p53* locus is more prevalent among xenografts than among real tumors or cell lines, and LOH at the *Rb* locus is equally prevalent in all three groups (Figure 6). Larger sample numbers are required to see whether these differences are statistically significant. Such studies of the prevalence of regions of LOH across model systems compared to real tumors may indicate systematic faults in the ability of model systems to reflect in vivo cancer biology and guide the use and development of appropriate models based on genetic organization.

SNP array analysis of cancer genomes provides a single platform for copy number and LOH analysis. As these arrays move to higher resolution (500K), accounting for the haplotype structure of the human genome in the analysis of these data will be of greater import. The methods described herein should be readily extensible to both the higher density arrays and to the increasingly detailed information describing the haplotype structure of the human genome. The software package, dChipSNP, is freely available at http://www.dchip.org.

## Materials and Methods

### Tumor samples and paired normals.

We used data from Early Access 10 K, Mapping 10 K, and 100 K SNP arrays (Affymetrix, Santa Clara, California, United States) (referred to as 10 K, 11 K and 100 K arrays, respectively) interrogating, respectively, 10,044, 11,555, and 116,204 SNP loci on all chromosomes except Y, with an average intermarker distance of 210 kb (11 K array) and 23.6 kb (100 K array) and average heterozygosity rate of 0.38 (11 K array) and 0.27 (100 K array) [7–9]. 10 K array data from paired tumor/normal lung and breast cancer cell lines were previously published [6,15]. 11 K data was obtained from prostate tumors, cell lines, and xenografts. 100 K data was obtained from prostate tumors, gliomas, and lung cancer cell lines, along with paired normal DNA from (respectively) seminal vesicles, normal brain, and EBV-transformed lymphocytes. Tumor DNA was isolated from frozen tissue having more than 90% tumor content. DNA preparation and genotyping were performed according to manufacturer's instructions. Insufficient DNA was available in the case of one prostate tumor, four EBV-transformed lymphocytes, and the paired normal for one glioma. In these cases 20 ng of DNA was subjected to whole-genome amplification [29] using the REPLI-g kit (Qiagen, Valencia, California, United States).

### Reference normal samples.

The heterozygosity rates for each SNP and the dependence information between the genotypes of neighboring SNPs were estimated from sets of normal samples; the haplotype correction was also performed against separate sets of normal samples (see Protocol S1). All these reference samples were from individuals unrelated to the tumor samples under evaluation. The estimated parameters are stored in genome information files available from the dChip website.

### Observed LOH calls from paired normal and tumor samples.

dChipSNP [12,28] was used to read CEL and TXT files containing the probe intensities and genotype calls (heterozygous AB, homozygous AA or BB, or missing genotype “No Call”) [30]. The paired normal and tumor data were combined to make LOH calls for each SNP marker: loss (AB in normal, AA or BB in tumor), retention (AB in normal and tumor, or No Call in normal and AB in tumor), noninformative (AA or BB in normal, and the same genotype or No Call in tumor), or conflict (e.g. AA in normal, and AB or BB in tumor). A HMM was used to infer copy numbers at each SNP position from the probe level intensity data of the SNP arrays [15]. The positions of the SNP markers, genes, and cytobands were based on Affymetrix annotation files (http://www.affymetrix.com) and the UCSC human genome assembly (http://genome.ucsc.edu).

## Supporting Information

Figure S1 Comparison of the NumHom Method to the HC/LD-HMM NumHom was applied using window sizes of 33 (with and without haplotype correction) and 50. Data are displayed as in Figure 3. Red stars indicate loci where NumHom breaks large regions of LOH into smaller ones, due to intervening heterozygous markers. Green boxes outline regions of NumHom-inferred LOH that are then regarded as retention in the haplotype correction.
(99 KB PDF)

Protocol S1 Supplemental Methods and Results (135 KB DOC)

Table S1 The Sensitivity and Specificity of the Basic HMM, LD-HMM, HC-HMM, and HC/LD-HMM The proportion of LOSS and RET markers identified in paired tumor/normal data, that were identified correctly in unpaired tumors in the 10 K dataset (A), 100 K training dataset (B), and 100 K validation dataset (C).
(53 KB DOC)

Table S2 The Sensitivity and Specificity of the Basic HMM and Haplotype-Corrected LD-HMM Ground truth was considered to be the results of a HMM applied to paired tumor/normal data.
(32 KB DOC)

Table S3 The Sensitivity and Specificity of the LD-HMM and HC/LD-HMM, Using Reference Samples from Alternative Ethnicities The number and proportion of SNP Markers in the 100 K Validation Dataset with LOSS or RET in Tumor/Normal Pairs, inferred as LOSS or RET by the LD-HMM (A) and HC/LD-HMM (B), using reference samples from alternative ethnicities.
(35 KB DOC)

Table S4 The Sensitivity and Specificity of the NumHom Method Using Different Threshold Window Sizes, Before and After Haplotype Correction (28 KB DOC)

Table S5 Sensitivity of the HC/LD-HMM for Regions of LOH The percentage of LOH regions identified in 10 K data from tumor/normal pairs that were also identified by the HC/LD-HMM applied to the unpaired tumors, according to the size of the region (A) or number of SNPs present (B).
(33 KB DOC)

Table S6 Most Common Regions of LOH in a Set of 34 Prostate Samples (36 KB DOC)

## Abbreviations

Het: heterozygous
HMM: hidden Markov model
Hom: homozygous
LD: linkage disequilibrium
LOH: loss of heterozygosity
LOSS: loss
Mb: megabase(s)
RET: retention
SNP: single-nucleotide polymorphism
TSG: tumor suppressor gene

## References

[pcbi-0020041-b001] Knudson AG (1971). Mutation and cancer: statistical study of retinoblastoma. Proc Natl Acad Sci U S A.

[pcbi-0020041-b002] Knudson AG (2001). Two genetic hits (more or less) to cancer. Nat Rev Cancer.

[pcbi-0020041-b003] Huang J, Wei W, Zhang J, Liu G, Bignell GR (2004). Whole genome DNA copy number changes identified by high density oligonucleotide arrays. Hum Genomics.

[pcbi-0020041-b004] McEvoy CR, Morley AA, Firgaira FA (2003). Evidence for whole chromosome 6 loss and duplication of the remaining chromosome in acute lymphoblastic leukemia. Genes Chromosomes Cancer.

[pcbi-0020041-b005] Girard L, Zochbauer-Muller S, Virmani AK, Gazdar AF, Minna JD (2000). Genome-wide allelotyping of lung cancer identifies new regions of allelic loss, differences between small cell lung cancer and non-small cell lung cancer, and loci clustering. Cancer Res.

[pcbi-0020041-b006] Janne PA, Li C, Zhao X, Girard L, Chen TH (2004). High-resolution single-nucleotide polymorphism array and clustering analysis of loss of heterozygosity in human lung cancer cell lines. Oncogene.

[pcbi-0020041-b007] Kennedy GC, Matsuzaki H, Dong S, Liu WM, Huang J (2003). Large-scale genotyping of complex DNA. Nat Biotechnol.

[pcbi-0020041-b008] Matsuzaki H, Dong S, Loi H, Di X, Liu G (2004). Genotyping over 100,000 SNPs on a pair of oligonucleotide arrays. Nature Methods.

[pcbi-0020041-b009] Matsuzaki H, Loi H, Dong S, Tsai YY, Fang J (2004). Parallel genotyping of over 10,000 SNPs using a one-primer assay on a high-density oligonucleotide array. Genome Res.

[pcbi-0020041-b010] Bignell GR, Huang J, Greshock J, Watt S, Butler A (2004). High-resolution analysis of DNA copy number using oligonucleotide microarrays. Genome Res.

[pcbi-0020041-b011] Hoque MO, Lee J, Begum S, Yamashita K, Engles JM (2003). High-throughput molecular analysis of urine sediment for the detection of bladder cancer by high-density single-nucleotide polymorphism array. Cancer Res.

[pcbi-0020041-b012] Lieberfarb ME, Lin M, Lechpammer M, Li C, Tanenbaum DM (2003). Genome-wide loss of heterozygosity analysis from laser capture microdissected prostate cancer using single nucleotide polymorphic allele (SNP) arrays and a novel bioinformatics platform dChipSNP. Cancer Res.

[pcbi-0020041-b013] Lindblad-Toh K, Tanenbaum DM, Daly MJ, Winchester E, Lui WO (2000). Loss-of-heterozygosity analysis of small-cell lung carcinomas using single-nucleotide polymorphism arrays. Nat Biotechnol.

[pcbi-0020041-b014] Mei R, Galipeau PC, Prass C, Berno A, Ghandour G (2000). Genome-wide detection of allelic imbalance using human SNPs and high-density DNA arrays. Genome Res.

[pcbi-0020041-b015] Zhao X, Li C, Paez JG, Chin K, Janne PA (2004). An integrated view of copy number and allelic alterations in the cancer genome using single nucleotide polymorphism arrays. Cancer Res.

[pcbi-0020041-b016] Goldberg EK, Glendening JM, Karanjawala Z, Sridhar A, Walker GJ (2000). Localization of multiple melanoma tumor-suppressor genes on chromosome 11 by use of homozygosity mapping-of-deletions analysis. Am J Hum Genet.

[pcbi-0020041-b017] Wong KK, Tsang YT, Shen J, Cheng RS, Chang YM (2004). Allelic imbalance analysis by high-density single-nucleotide polymorphic allele (SNP) array with whole genome amplified DNA. Nucleic Acids Res.

[pcbi-0020041-b018] Burge C, Karlin S (1997). Prediction of complete gene structures in human genomic DNA. J Mol Biol.

[pcbi-0020041-b019] Churchill GA (1989). Stochastic models for heterogeneous DNA sequences. Bull Math Biol.

[pcbi-0020041-b020] Durbin R, Eddy S, Krogh A, Mitchison G (1999). Biological sequence analysis: Probabilistic models of proteins and nucleic acids.

[pcbi-0020041-b021] Lander ES, Green P (1987). Construction of multilocus genetic linkage maps in humans. Proc Natl Acad Sci U S A.

[pcbi-0020041-b022] Lange K (2002). Mathematical and statistical methods for genetic analysis.

[pcbi-0020041-b023] Fridlyand J, Snijders AM, Pinkel D, Albertson DG, Jain AN (2004). Hidden Markov models approach to the analysis of array CGH data. J Multivar Anal.

[pcbi-0020041-b024] Miller BJ, Wang D, Krahe R, Wright FA (2003). Pooled analysis of loss of heterozygosity in breast cancer: A genome scan provides comparative evidence for multiple tumor suppressors and identifies novel candidate regions. Am J Hum Genet.

[pcbi-0020041-b025] Newton MA, Gould MN, Reznikoff CA, Haag JD (1998). On the statistical analysis of allelic-loss data. Stat Med.

[pcbi-0020041-b026] Liu N, Sawyer SL, Mukherjee N, Pakstis AJ, Kidd JR (2004). Haplotype block structures show significant variation among populations. Genet Epidemiol.

[pcbi-0020041-b027] Altshuler D, Brooks LD, Chakravarti A, Collins FS, Daly MJ (2005). A haplotype map of the human genome. Nature.

[pcbi-0020041-b028] Lin M, Wei LJ, Sellers WR, Lieberfarb M, Wong WH (2004). dChipSNP: Significance curve and clustering of SNP-array-based loss-of-heterozygosity data. Bioinformatics.

[pcbi-0020041-b029] Paez JG, Lin M, Beroukhim R, Lee JC, Zhao X (2004). Genome coverage and sequence fidelity of phi29 polymerase-based multiple strand displacement whole genome amplification. Nucleic Acids Res.

[pcbi-0020041-b030] Liu WM, Di X, Yang G, Matsuzaki H, Huang J (2003). Algorithms for large-scale genotyping microarrays. Bioinformatics.

