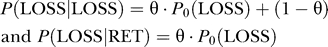# Correction: Inferring Loss-of-Heterozygosity from Unpaired Tumors Using High-Density Oligonucleotide SNP Arrays

**DOI:** 10.1371/journal.pcbi.0030040

**Published:** 2007-02-23

**Authors:** Rameen Beroukhim, Ming Lin, Yuhyun Park, Ke Hao, Xiaojun Zhao, Levi A Garraway, Edward A Fox, Ephraim P Hochberg, Ingo K Mellinghoff, Matthias D Hofer, Aurelien Descazeaud, Mark A Rubin, Matthew Meyerson, Wing Hung Wong, William R Sellers, Cheng Li

In *PLoS Computational Biology,* volume 2, issue 5: doi:10.1371/journal.pcbi.0020041

In the subsection “Initial probabilities” of the Results section, the first sentence was incorrect and should say:

These probabilities, denoted by *P*_0_(RET) and *P*_0_(LOSS) = 1 − *P*_0_(RET), specify the probabilities of RET and LOSS for the p-terminal marker on a chromosome.

The second line of Equation 1 was incorrect. The correct equation is: