# Supplementary material for: Inferring Loss-of-Heterozygosity from Unpaired Tumors Using High-Density Oligonucleotide SNP Arrays
Source: PLoS Comput Biol. 2006 May 12;2(5):e41. doi: 10.1371/journal.pcbi.0020041 (PMC1458964; doi:10.1371/journal.pcbi.0020041)
Supplement: Supplementary file 2 [file pcbi.0020041.sd001.doc]

SUPPLEMENTAL METHODS

## Reference normal samples

The heterozygosity rates for each SNP and the dependence information between the genotypes of neighboring SNPs were estimated, in the case of the 10K and 11K arrays, from data obtained from normal DNA, from 59 participants in a bone marrow transplant study (Hochberg et al, in preparation), the 60 parents in the CEPH set of family trios, and 42 others (and C. Rosenow, pers. comm.). Ethnicity data was not recorded for the bone marrow transplant study, but the majority of participants were likely Caucasian; all other samples were Caucasian. For the 100K array, these data were obtained from the 60 parents in the CEPH set, available from

<https://www.affymetrix.com/support/technical/sample_data/hapmap_trio_data.affx>. The 60 parents of the YOR (African) set and the 89 members of the JHC (East Asian) set of HapMap individuals were used as reference normals in tests of mismatching the ethnicities of tumors and controls. For the haplotype correction, regions of inferred LOH were compared against a reference set of normal samples. In the case of the 10K array, this reference set contained 31 samples taken from the bone marrow transplant study described above. In the case of the 11K array, this reference set contained 31 samples. Twenty-three of these were normal control data previously published in a breast cancer study [1] and 8 represented data, obtained from various sources, from Caucasians without cancer. In the case of the 100K array, the reference set included DNA from prostates of 10 men who underwent prostatectomy for benign prostatic hypertrophy, 5 people without cancer, and normal brain tissue from 3 people who had gliomas for which tumor was insufficiently enriched for LOH to be observed. Of these, 11 samples were from Caucasians, 1 from a Hispanic, and ethnicity data was not recorded for the other 6.

SUPPLEMENTAL RESULTS

## HMM for inferring LOH in paired samples at noninformative regions

To test the sensitivity and specificity of the basic HMM and haplotype-corrected LD-HMM, we required a way of assigning RET and LOSS states to noninformative markers in the paired analysis (see Results). To ensure that the method we used to assign these states was not biasing our results, we took alternative approaches and compared the results they gave. In addition to assigning to noninformative markers the LOH states of the nearest flanking informative markers (as described in Results), we also developed an HMM to infer LOH in these noninformative regions in the paired tumor/normal datasets. For this HMM, the unobserved LOH states remained LOSS and RET, but the observed data were the observed LOSS, RET, Noninformative, and Conflict calls. The initial and transition probabilities were estimated as in the basic HMM for unpaired tumors. The emission probability of observing a LOSS call given an underlying LOSS state and the probability of observing a RET call given an underlying RET state were set to be (1 – error rate) to reflect genotyping and mapping errors. Noninformative and Conflict calls were emitted with probability 1 regardless of the underlying LOH state. The HMM was computed using the Forward-Backward algorithm, and LOSS calls were made when the probability of LOSS was greater than 0.5. We used this HMM to recalculate the sensitivity and specificity of the basic HMM and haplotype-corrected LD-HMM, using the LOH states assigned by the HMM applied to paired data as ground truth. This analysis gave results similar to those obtained when noninformative markers were assigned the same LOH states as the nearest flanking informative markers (Supplemental Table 2, compare to Supplemental Table 1).

SUPPLEMENTAL TABLES

**Supplemental Table 1.**

|  | Observed | | Noninformative flanked by | | Overall | |
| --- | --- | --- | --- | --- | --- | --- |
|  | LOSS(17922) | RET(16364) | LOSS(41815) | RET(47614) | LOSS(59737) | RET(63978) |
| Basic HMM | 97.7% | 97.5% | 99.7% | 93.2% | 99.1% | 94.3% |
| LD-HMM | 97.6% | 97.9% | 99.6% | 94.6% | 99.0% | 95.4% |
| HC-HMM | 97.0% | 97.7% | 98.9% | 95.4% | 98.3% | 96.0% |
| HC/LD-HMM | 96.9% | 98.0% | 98.8% | 96.0% | 98.2% | 96.5% |

B.

|  | Observed | | Noninformative flanked by | | Overall | |
| --- | --- | --- | --- | --- | --- | --- |
|  | LOSS(20961) | RET(62509) | LOSS(88805) | RET(268009) | LOSS(109766) | RET(330518) |
| Basic HMM | 99.0% | 98.8% | 99.9% | 90.7% | 99.8% | 92.2% |
| LD-HMM | 98.8% | 99.4% | 99.7% | 96.9% | 99.6% | 97.4% |
| HC-HMM | 98.7% | 99.2% | 99.6% | 97.4% | 99.4% | 97.7% |
| HC/LD-HMM | 98.8% | 99.4% | 99.7% | 98.9% | 99.5% | 99.0% |

C.

|  | Observed | | Noninformative flanked by | | Overall | |
| --- | --- | --- | --- | --- | --- | --- |
|  | LOSS(35584) | RET(149219) | LOSS(135823) | RET(552938) | LOSS(171407) | RET(702157) |
| Basic HMM | 97.6% | 99.2% | 99.7% | 92.5% | 99.3% | 94.0% |
| LD-HMM | 97.4% | 99.6% | 99.6% | 97.6% | 99.1% | 98.0% |
| HC-HMM | 97.2% | 99.5% | 99.4% | 98.1% | 99.0% | 98.4% |
| HC/LD-HMM | 97.0% | 99.6% | 99.1% | 99.2% | 98.7% | 99.3% |

**Supplemental Table 2.**

|  | 10K training data | | 100K training data | | 100K validation data | |
| --- | --- | --- | --- | --- | --- | --- |
|  | Sensitivity  LOSS(64805) | Specificity  RET(70771) | Sensitivity  LOSS(115064) | Specificity  RET(336896) | Sensitivity  LOSS(179590) | Specificity  RET(724330) |
| Basic HMM | 99.2% | 91.8% | 99.9% | 91.8% | 99.7% | 93.7% |
| LD-HMM | 99.2% | 93.1% | 99.8% | 96.9% | 99.6% | 97.8% |
| HC-HMM | 98.2% | 94.0% | 99.5% | 97.4% | 99.3% | 98.3% |
| HC/LD-HMM | 98.1% | 94.7% | 99.7% | 98.6% | 99.1% | 99.3% |

**Supplemental Table 3.**

A. LD-HMM

|  | | CEPH references | | JHC references | | Yoruba references | |
| --- | --- | --- | --- | --- | --- | --- | --- |
|  |  | LOSS | RET | LOSS | RET | LOSS | RET |
| Tumor/normal pairs | LOSS  (109766) | 109294  (99.6%) | 472  (0.4%) | 109289  (99.6%) | 477  (0.4%) | 109379  (99.6) | 387  (0.4%) |
|  | RET  (330518) | 8729  (2.6%) | 321789  (97.4%) | 9828  (3.0%) | 320690  (97.0%) | 20346  (6.2%) | 310172  (93.8%) |

B. HC/LD-HMM*

|  | | Caucasian references | | JHC references | |
| --- | --- | --- | --- | --- | --- |
|  |  | LOSS | RET | LOSS | RET |
| Tumor/normal pairs | LOSS  (109766) | 109261  (99.5%) | 505  (0.5%) | 109281  (99.6%) | 485  (0.4%) |
|  | RET  (330518) | 3369  (1.0%) | 330518  (99.0%) | 5725  (1.7%) | 324793  (98.3%) |

*CEPH references were used to determine SNP-specific heterozygosity rates and dependencies between neighboring SNPs; a different set of either Caucasian or JHC references were used for haplotype correction.

**Supplemental Table 4.**

|  |  | Without HC | | With HC | |
| --- | --- | --- | --- | --- | --- |
| Number of homozygous loci | Frequency in normal samples* | Sensitivity | Specificity | Sensitivity | Specificity |
| 17 | 0.05 | 99.7 | 73.4 | 94.3 | 99.3 |
| 33 | 0.01 | 99.2 | 90.4 | 94.3 | 99.6 |
| 50 | 0.002 | 98.7 | 96.7 | 94.2 | 99.8 |
| LD-HMM** | - | 99.6 | 97.4 | 99.5 | 99.0 |

*The frequency with which a window of this size, containing only homozygous markers, occurs in the normal reference set

**The performance of the LD-HMM is listed for comparison

**Supplemental Table 5.**

A.

| Size of region (Mb) | Number of regions (per cent of total) | Number of informative SNPs (mean ± sd)* | Proportion identified by tumor only |
| --- | --- | --- | --- |
|  1 | 5 (1.6%) | 4.2 ± 1.6 | 40.0% |
| 1 – 3 | 14 (4.4%) | 3.9 ± 1.3 | 21.4% |
| 3-10 | 47 (14.8%) | 7.3 ± 4.3 | 59.6% |
| > 10 | 251 (79.2%) | 68 ± 57 | 96.8% |
| All | 317 (100%) | 55 ± 57 | 87.1% |

B.

| Number of SNPs in region | Number of regions (per cent of total) | Number of informative SNPs (mean ± sd)* | Proportion identified by tumor only |
| --- | --- | --- | --- |
| 1 – 40 | 74 (23.3%) | 6.0 ± 3.6 | 52.7% |
| 41 – 100 | 70 (22.1%) | 18.4 ± 7.4 | 91.4% |
| 100+ | 173 (54.6%) | 91 ± 54 | 100% |
| All | 317 (100%) | 55 ± 57 | 87.1% |

***”**sd” represents standard deviation

**Supplemental Table 6.**

| Chromosome arm | Minimally deleted region* | Putative tumor suppressor gene | LOH prevalence (%)** | Reported prevalence*** |
| --- | --- | --- | --- | --- |
| 1q | 1q44 |  | 38 | 26 |
| 5q | 5q15-21.3 |  | 47 | 57 |
| 6q | 6q15-16.1 |  | 41 | 48 |
| 8p | 8p12-22 | NKX3.1 (8p21.2) | 81 | 43 |
| 10q | 10q23.2-24.32 | PTEN (10q23.2) | 34 | 43 |
| 12p | 12p12.3-13.31 | p27 (12p13.1) | 31 | 38 |
| 13q | 13q14.11-14.2 | Rb (13q14.2) | 53 | 61 |
| 16q | 16q12.1-21 |  | 44 | 48 |
| 17p | 17p12-13.3 | p53 (17p13.1) | 63 | 49 |
| 18q | 18q22.3-23 |  | 63 | 45 |

*Region with >90% of the peak LOH probablity score on the chromosome

**Across our samples, at the region of peak LOH prevalence

***Reported in metastatic samples [2]

**References**

1. Allinen M, Beroukhim R, Cai L, Brennan C, Lahti-Domenici J, et al. (2004) Molecular characterization of the tumor microenvironment in breast cancer. Cancer Cell 6: 17-32.

2. Kibel AS, Faith DA, Bova GS, Isaacs WB (2000) Loss of heterozygosity at 12P12-13 in primary and metastatic prostate adenocarcinoma. J Urol 164: 192-196.
