# Supplementary material for: Inferring Loss-of-Heterozygosity from Unpaired Tumors Using High-Density Oligonucleotide SNP Arrays
Source: PLoS Comput Biol. 2006 May 12;2(5):e41. doi: 10.1371/journal.pcbi.0020041 (PMC1458964; doi:10.1371/journal.pcbi.0020041)
Supplement: Supplementary file 3 [file pcbi.0020041.st001.doc]

**Supplemental Table 1.**

|  | Observed | | Noninformative flanked by | | Overall | |
| --- | --- | --- | --- | --- | --- | --- |
|  | LOSS(17922) | RET(16364) | LOSS(41815) | RET(47614) | LOSS(59737) | RET(63978) |
| Basic HMM | 97.7% | 97.5% | 99.7% | 93.2% | 99.1% | 94.3% |
| LD-HMM | 97.6% | 97.9% | 99.6% | 94.6% | 99.0% | 95.4% |
| HC-HMM | 97.0% | 97.7% | 98.9% | 95.4% | 98.3% | 96.0% |
| HC/LD-HMM | 96.9% | 98.0% | 98.8% | 96.0% | 98.2% | 96.5% |

B.

|  | Observed | | Noninformative flanked by | | Overall | |
| --- | --- | --- | --- | --- | --- | --- |
|  | LOSS(20961) | RET(62509) | LOSS(88805) | RET(268009) | LOSS(109766) | RET(330518) |
| Basic HMM | 99.0% | 98.8% | 99.9% | 90.7% | 99.8% | 92.2% |
| LD-HMM | 98.8% | 99.4% | 99.7% | 96.9% | 99.6% | 97.4% |
| HC-HMM | 98.7% | 99.2% | 99.6% | 97.4% | 99.4% | 97.7% |
| HC/LD-HMM | 98.8% | 99.4% | 99.7% | 98.9% | 99.5% | 99.0% |

C.

|  | Observed | | Noninformative flanked by | | Overall | |
| --- | --- | --- | --- | --- | --- | --- |
|  | LOSS(35584) | RET(149219) | LOSS(135823) | RET(552938) | LOSS(171407) | RET(702157) |
| Basic HMM | 97.6% | 99.2% | 99.7% | 92.5% | 99.3% | 94.0% |
| LD-HMM | 97.4% | 99.6% | 99.6% | 97.6% | 99.1% | 98.0% |
| HC-HMM | 97.2% | 99.5% | 99.4% | 98.1% | 99.0% | 98.4% |
| HC/LD-HMM | 97.0% | 99.6% | 99.1% | 99.2% | 98.7% | 99.3% |
