# Supplementary material for: Inferring Loss-of-Heterozygosity from Unpaired Tumors Using High-Density Oligonucleotide SNP Arrays
Source: PLoS Comput Biol. 2006 May 12;2(5):e41. doi: 10.1371/journal.pcbi.0020041 (PMC1458964; doi:10.1371/journal.pcbi.0020041)
Supplement: Supplementary file 4 [file pcbi.0020041.st002.doc]

**Supplemental Table 2.**

|  | 10K training data | | 100K training data | | 100K validation data | |
| --- | --- | --- | --- | --- | --- | --- |
|  | Sensitivity  LOSS(64805) | Specificity  RET(70771) | Sensitivity  LOSS(115064) | Specificity  RET(336896) | Sensitivity  LOSS(179590) | Specificity  RET(724330) |
| Basic HMM | 99.2% | 91.8% | 99.9% | 91.8% | 99.7% | 93.7% |
| LD-HMM | 99.2% | 93.1% | 99.8% | 96.9% | 99.6% | 97.8% |
| HC-HMM | 98.2% | 94.0% | 99.5% | 97.4% | 99.3% | 98.3% |
| HC/LD-HMM | 98.1% | 94.7% | 99.7% | 98.6% | 99.1% | 99.3% |
