# Supplementary material for: Inferring Loss-of-Heterozygosity from Unpaired Tumors Using High-Density Oligonucleotide SNP Arrays
Source: PLoS Comput Biol. 2006 May 12;2(5):e41. doi: 10.1371/journal.pcbi.0020041 (PMC1458964; doi:10.1371/journal.pcbi.0020041)
Supplement: Supplementary file 5 [file pcbi.0020041.st003.doc]

**Supplemental Table 3.**

A. LD-HMM

|  | | CEPH references | | JHC references | | Yoruba references | |
| --- | --- | --- | --- | --- | --- | --- | --- |
|  |  | LOSS | RET | LOSS | RET | LOSS | RET |
| Tumor/normal pairs | LOSS  (109766) | 109294  (99.6%) | 472  (0.4%) | 109289  (99.6%) | 477  (0.4%) | 109379  (99.6) | 387  (0.4%) |
|  | RET  (330518) | 8729  (2.6%) | 321789  (97.4%) | 9828  (3.0%) | 320690  (97.0%) | 20346  (6.2%) | 310172  (93.8%) |

B. HC/LD-HMM*

|  | | Caucasian references | | JHC references | |
| --- | --- | --- | --- | --- | --- |
|  |  | LOSS | RET | LOSS | RET |
| Tumor/normal pairs | LOSS  (109766) | 109261  (99.5%) | 505  (0.5%) | 109281  (99.6%) | 485  (0.4%) |
|  | RET  (330518) | 3369  (1.0%) | 330518  (99.0%) | 5725  (1.7%) | 324793  (98.3%) |

*CEPH references were used to determine SNP-specific heterozygosity rates and dependencies between neighboring SNPs; a different set of either Caucasian or JHC references were used for haplotype correction.
