# Supplementary material for: Inferring Loss-of-Heterozygosity from Unpaired Tumors Using High-Density Oligonucleotide SNP Arrays
Source: PLoS Comput Biol. 2006 May 12;2(5):e41. doi: 10.1371/journal.pcbi.0020041 (PMC1458964; doi:10.1371/journal.pcbi.0020041)
Supplement: Supplementary file 6 [file pcbi.0020041.st004.doc]

**Supplemental Table 4.**

|  |  | Without HC | | With HC | |
| --- | --- | --- | --- | --- | --- |
| Number of homozygous loci | Frequency in normal samples* | Sensitivity | Specificity | Sensitivity | Specificity |
| 17 | 0.05 | 99.7 | 73.4 | 94.3 | 99.3 |
| 33 | 0.01 | 99.2 | 90.4 | 94.3 | 99.6 |
| 50 | 0.002 | 98.7 | 96.7 | 94.2 | 99.8 |
| LD-HMM** | - | 99.6 | 97.4 | 99.5 | 99.0 |

*The frequency with which a window of this size, containing only homozygous markers, occurs in the normal reference set

**The performance of the LD-HMM is listed for comparison
