# Supplementary material for: Inferring Loss-of-Heterozygosity from Unpaired Tumors Using High-Density Oligonucleotide SNP Arrays
Source: PLoS Comput Biol. 2006 May 12;2(5):e41. doi: 10.1371/journal.pcbi.0020041 (PMC1458964; doi:10.1371/journal.pcbi.0020041)
Supplement: Supplementary file 7 [file pcbi.0020041.st005.doc]

**Supplemental Table 5.**

A.

| Size of region (Mb) | Number of regions (per cent of total) | Number of informative SNPs (mean ± sd)* | Proportion identified by tumor only |
| --- | --- | --- | --- |
|  1 | 5 (1.6%) | 4.2 ± 1.6 | 40.0% |
| 1 – 3 | 14 (4.4%) | 3.9 ± 1.3 | 21.4% |
| 3-10 | 47 (14.8%) | 7.3 ± 4.3 | 59.6% |
| > 10 | 251 (79.2%) | 68 ± 57 | 96.8% |
| All | 317 (100%) | 55 ± 57 | 87.1% |

B.

| Number of SNPs in region | Number of regions (per cent of total) | Number of informative SNPs (mean ± sd)* | Proportion identified by tumor only |
| --- | --- | --- | --- |
| 1 – 40 | 74 (23.3%) | 6.0 ± 3.6 | 52.7% |
| 41 – 100 | 70 (22.1%) | 18.4 ± 7.4 | 91.4% |
| 100+ | 173 (54.6%) | 91 ± 54 | 100% |
| All | 317 (100%) | 55 ± 57 | 87.1% |

***”**sd” represents standard deviation
