# Supplementary material for: Inferring Loss-of-Heterozygosity from Unpaired Tumors Using High-Density Oligonucleotide SNP Arrays
Source: PLoS Comput Biol. 2006 May 12;2(5):e41. doi: 10.1371/journal.pcbi.0020041 (PMC1458964; doi:10.1371/journal.pcbi.0020041)
Supplement: Supplementary file 8 [file pcbi.0020041.st006.doc]

**Supplemental Table 6.**

| Chromosome arm | Minimally deleted region* | Putative tumor suppressor gene | LOH prevalence (%)** | Reported prevalence*** |
| --- | --- | --- | --- | --- |
| 1q | 1q44 |  | 38 | 26 |
| 5q | 5q15-21.3 |  | 47 | 57 |
| 6q | 6q15-16.1 |  | 41 | 48 |
| 8p | 8p12-22 | NKX3.1 (8p21.2) | 81 | 43 |
| 10q | 10q23.2-24.32 | PTEN (10q23.2) | 34 | 43 |
| 12p | 12p12.3-13.31 | p27 (12p13.1) | 31 | 38 |
| 13q | 13q14.11-14.2 | Rb (13q14.2) | 53 | 61 |
| 16q | 16q12.1-21 |  | 44 | 48 |
| 17p | 17p12-13.3 | p53 (17p13.1) | 63 | 49 |
| 18q | 18q22.3-23 |  | 63 | 45 |

*Region with >90% of the peak LOH probablity score on the chromosome

**Across our samples, at the region of peak LOH prevalence

***Reported in metastatic samples [1]

**References**

1. Kibel AS, Faith DA, Bova GS, Isaacs WB (2000) Loss of heterozygosity at 12P12-13 in primary and metastatic prostate adenocarcinoma. J Urol 164: 192-196.
